# Value of Ethnicity or Race in More Accurate Prediction of Future Outcome in Couples with Recurrent Miscarriages

**DOI:** 10.1007/s43032-022-00942-x

**Published:** 2022-04-28

**Authors:** Marie-Louise van der Hoorn, Lisa Lashley

**Affiliations:** Department of Gynaecology and Obstetrics, Leiden University Medical Center, 2333 ZA Leiden, the Netherlands

**Keywords:** Ethnicity, Recurrent pregnancy loss, Prediction

## Abstract

This special issue of Reproductive Sciences is focusing on ethnic health disparity and its impact on (fe)male reproduction. Indeed, studies regarding underlying mechanisms, interventions and prognosis in reproduction are underexposed for the non-White male and female. Here, we call for documentation of race and ethnicity in the analysis and management of couples with recurrent pregnancy loss.

To the Editor:

This special issue of Reproductive Sciences is focusing on ethnic health disparity and its impact on (fe)male reproduction. Indeed, studies regarding underlying mechanisms, interventions and prognosis in reproduction are underexposed for the non-White male and female. Here, we call for documentation of race and ethnicity in the analysis and management of couples with recurrent pregnancy loss (RPL).

Recurrent pregnancy loss is defined as the loss of two or more conceptions before the 24^th^ week of gestation. It is a poorly understood condition that comes with many uncertainties. A better understanding of contributing factors is necessary to provide answers to the couples and to improve their pregnancy outcomes. Currently, there is hardly any focus on race, or ethnicity, and its contribution to the development of RPL. For some reproductive disorders, it is clear that there is differential prevalence in one ethnic or racial group versus the other [1]. Also for RPL, it was shown recently that Black ethnicity is associated with a higher risk of miscarriage compared to White ethnicity [2]. Whether the miscarriage risk differs across nations and other ethnic groups is currently however under-documented.

For couples with RPL, the question regarding chance of a future successful pregnancy is utmost important. Even when aetiological mechanisms are not fully elucidated, well-developed and validated prediction models may provide adequate estimates of future pregnancy outcomes [3].

In today’s clinical practice, two prediction models for couples with unexplained RPL [4, 5] are used and recommended by international clinical guidelines. As a result of changing definition and diagnostic investigations for RPL, this could affect the predictive performance in other population [6]. Moreover, these models are based on only two predictors: the number of previous pregnancy losses and maternal age. We suggested recently that the prediction of a subsequent ongoing pregnancy in couples with RPL could improve when taking additional candidate predictors into account [7].

Female and male of racial and ethnic minority groups may be genetically predisposed to worse outcomes, independent of other clinical risk factors. In addition, race and ethnicity may serve as a surrogate for known risk factors of RPL, such as age, obesity, uterine abnormalities, smoking or other environmental risk factors (8). This implies that race or ethnicity, of both female as male, could represent a suitable predictor candidate to improve predictive ability of the prediction model. More research is necessary to verify this statement. However, to investigate these variables we must start with the documentation of race and ethnicity in our analysis and management in couples with RPL. Hopefully, this will add to the knowledge on RPL and add to Reproductive Science’ aim of health equity.

## Data Availability

Not applicable.
